# A cluster randomised feasibility study of an adolescent incentive intervention to increase uptake of HPV vaccination

**DOI:** 10.1038/bjc.2017.284

**Published:** 2017-08-22

**Authors:** Alice S Forster, Victoria Cornelius, Lauren Rockliffe, Laura AV Marlow, Helen Bedford, Jo Waller

**Affiliations:** 1 Research Department of Behavioural Science and Health, UCL, Gower Street, London WC1E 6BT, UK; 2 Imperial Clinical Trials Unit, School of Public Health, Imperial College London, Stadium House, 68 Wood Lane, London W12 7RH, UK; 3 UCL Great Ormond Street Institute of Child Health, 30 Guilford Street, London WC1N 1EH, UK

**Keywords:** vaccination, reward, adolescent, papillomavirus vaccines, motivation, prevention

## Abstract

**Background:**

Uptake of human papillomavirus (HPV) vaccination is suboptimal among some groups. We aimed to determine the feasibility of undertaking a cluster randomised controlled trial (RCT) of incentives to improve HPV vaccination uptake by increasing consent form return.

**Methods:**

An equal-allocation, two-arm cluster RCT design was used. We invited 60 London schools to participate. Those agreeing were randomised to either a standard invitation or incentive intervention arm, in which Year 8 girls had the chance to win a £50 shopping voucher if they returned a vaccination consent form, regardless of whether consent was provided. We collected data on school and parent participation rates and questionnaire response rates. Analyses were descriptive.

**Results:**

Six schools completed the trial and only 3% of parents opted out. The response rate was 70% for the girls’ questionnaire and 17% for the parents’. In the intervention arm, 87% of girls returned a consent form compared with 67% in the standard invitation arm. The proportion of girls whose parents gave consent for vaccination was higher in the intervention arm (76%) than the standard invitation arm (61%).

**Conclusions:**

An RCT of an incentive intervention is feasible. The intervention may improve vaccination uptake but a fully powered RCT is needed.

Uptake of the human papillomavirus (HPV) vaccine for the prevention of HPV-related cancers in England is good, with 87% of the routine cohort of girls receiving the first of two doses in 2015/2016 (Public Health England, 2016). However, there is large geographical variation, with uptake as low as 68% in some areas (Public Health England, 2016) and ethnic disparities in uptake that remain irrespective of deprivation level (Fisher *et al*, 2013a,b; Bowyer *et al*, 2014). High uptake will offer herd protection to unvaccinated individuals who will have sex with women. There is limited evidence about what can be done to improve HPV vaccination uptake in a UK setting (Fu *et al*, 2014; Walling *et al*, 2016).

In the English programme, informed consent is required for vaccination, which is most commonly provided by each girl’s legal guardian (usually a parent) (Public Health England, 2013). In most instances, schools ask girls to hand deliver a consent form to their parent for them to sign and then to hand deliver it back to school. Consent forms must be returned regardless of whether consent to vaccination is granted. Audit findings suggests that around 60% of consent forms are returned without prompting, granting consent to vaccination; half of the remaining 40% are returned, consenting to vaccination, if they are chased by an immunisation nurse (Bentley, 2015). (This 80% uptake is lower than the current national uptake figures, which may be due to the audit data pertaining to particular geographical areas, as well as being collected in a different academic year). This suggests that interventions to promote consent form return (distinct from interventions to promote vaccination receipt) may result in improved vaccination uptake. Such an intervention may have an additional benefit of reducing immunisation nurse workload. Higher rates of consent form return have been attributed to persistent efforts by school and immunisation staff to pursue adolescents who have not returned their consent form (Perman *et al*, 2017). However, some school staff do not have the resources to pursue unreturned consent forms (Batista Ferrer *et al*, 2015). Interventions to improve consent form return therefore need to be undemanding to deliver; incentivising consent form return may be a suitable intervention.

Financial incentives provided contingent on vaccination receipt have been used with varying effect (Sutherland *et al*, 2008; Bassani *et al*, 2013; Wigham *et al*, 2014; Betsch *et al*, 2015; Dempsey and Zimet, 2015; Jarrett *et al*, 2015; Mantzari *et al*, 2015); however, no trial has reported on incentivising consent form return. There are a number of possible mechanisms by which such a strategy might improve consent form return. The perceived value of returning a consent form may be increased (Camerer and Hogarth, 1999; Dawes, 1999; Hertwig and Ortmann, 2001; Rosenstock *et al*, 1988; Lopes, 1994; Zwick *et al*, 1999), it might improve memory to return the form (Kang *et al*, 2009; Marteau, 2010) or there may be a role for the fear of missing out (FOMO) on something that one’s friends are experiencing (Przybylski *et al*, 2013). The acceptability of incentives to improve health behaviours varies, with variation by the incentive type, how effective it is, the behaviour being targeted, as well as socio-demographic characteristics of the individuals being asked (NICE, 2010; Promberger *et al*, 2012; Marshall *et al*, 2014; Morgan *et al*, 2015; Giles *et al*, 2016a,b; Adams *et al*, 2016; McNaughton *et al*, 2016). Concern has been expressed that parents’ choice may be undermined (Gardner *et al*, 2010; Mantzari *et al*, 2015), with parents being prevented from making informed decisions about vaccination, although it may result in parents being forced to engage with the decision.

Before testing the efficacy of any incentive intervention in a randomised controlled trial (RCT), one must ensure that the RCT is feasible. Here we present findings of a cluster randomised feasibility trial of an adolescent incentive intervention to increase uptake of HPV vaccination among girls, by promoting consent form return. The key objectives were as follows: (1) to describe the feasibility of the future RCT by assessing participation rates, and data quality and completeness, and (2) to generate proof-of-concept evidence of the effect of the intervention on consent form return rates and vaccination uptake, and any possible unintended consequences of the intervention and mechanisms of action.

## Materials and methods

We conducted an equal allocation, two-arm cluster randomised feasibility study in the London Boroughs (districts) of Enfield, Lambeth and Southwark, with schools within these boroughs as clusters. The demographic and vaccination profile of these boroughs has been described previously in a detailed protocol (Forster *et al*, 2017). We collected data from school staff, participating girls and their parents between July 2016 and January 2017. Schools in participating boroughs were eligible for inclusion if they had female Year 8 students (12–13 years old), and were identified through local authority school lists.

### Interventions

The intervention and its development has been described in detail elsewhere (Forster *et al*, 2017) and was delivered at the cluster-level. The intervention was aimed at Year 8 girls who were due to be invited to receive the HPV vaccine. A standard invitation arm comprised girls being provided with an information leaflet about the HPV vaccine and a consent form from the school, which they were asked to hand deliver to their parents and return before a prescribed date. Girls in the incentive intervention arm received the standard invitation. They were also told by their form tutor and in a letter that they would be eligible to be entered into a prize draw to win a £50 Love2Shop voucher (shopping vouchers redeemable at over 20 000 UK high street shops) if they returned their consent form, signed by a legal guardian, before a prescribed date. Eligibility for entry into the prize draw was dependent on consent form return only, not vaccine receipt. Girls were eligible regardless of whether their returned form granted consent or not. All girls who returned their consent form were entered into a prize draw for each school, with girls having a 1 in 10 chance of winning. The draw was made following the first dose of the HPV vaccine, as all consent forms had to be returned by this point.

### Procedure and outcomes

We approached all secondary schools in participating boroughs initially via email and then by telephone.

Participating schools informed parents that they could opt out of the study. We collected data on schools’ and parents’ willingness to participate in the study. A statistician used computer generated random numbers to allocate schools into each arm using blocked randomisation. Schools were not blind to allocation. We recorded publically available information on school size, whether single sex/co-educational and religious affiliation for participating and non-participating schools.

We sent schools randomised to the incentive intervention arm the intervention letter to give to their eligible students and form tutors told their female students that they could be eligible for an incentive. Concurrently, parents in both arms were asked to consent to their daughter having the HPV vaccine as part of the routine immunisation programme, as per the standard invitation intervention, with vaccination occurring in the next 2–3 weeks.

School administrative staff assigned an anonymised identifier to participating girls and collected data on whether participating girls returned their consent forms and whether consent was given for vaccination. They provided these data to researchers anonymised, along with a marker of deprivation (index of multiple deprivation quintile, IMD), based on postcode, for each girl.

Teachers asked girls to complete a questionnaire during school hours within a week of vaccination day, and the school sent parents a questionnaire to complete. Parent questionnaires were returned directly to researchers in postage paid envelopes. Both questionnaires were linked to each girl’s anonymous identifier. We collected data on response rates to questionnaires and data completeness. The girls’ questionnaire assessed possible mechanisms of action of the intervention including: the incentive increasing motivation to return the consent form, the incentive improving memory to return the consent form, the incentive making it more likely that the consent form is given to parents promptly, the incentive increases the perceived value of returning the consent form and FOMO (using Przybylski *et al*, 2013). Girls in the incentive arm were also asked about possible unintended consequences of the intervention (i.e., would they return consent forms in the future without incentive?). The parents’ questionnaire assessed parents’ attitudes towards the incentive offered and whether parents made an informed decision about the vaccine using questions as described by Mantzari *et al* (2015). Girls reported their religion (based on Office for National Statistics, 2011), strength of any religious faith (European Social Survey) and migration status (whether they and their parents were born in the United Kingdom; adapted from Marlow *et al* (2015)). Parents reported their daughter’s ethnicity (using Office for National Statistics (2011)). Full details of the questionnaire items are provided in Supplementary Material. We generated items for the present study, unless specified.

The sample size was based on being able to estimate feasibility outcomes. On average, in participating boroughs there were around 100 girls in Year 8 per school. With 600 girls it would be possible to estimate the participation rates, questionnaire response rates and acceptability of the trial methods, with an unadjusted precision of the 95% confidence intervals (95% CI) to at least ±5 percentage points, assuming the most statistically conservative scenario (Forster *et al*, 2017). It was not an objective of this feasibility study to estimate the intracluster correlation as the number of clusters would be too small.

### Analysis

The analyst was blind to group allocation until analysis was complete. Where participants had returned a questionnaire, missing items within scales were replaced with the mean for that item if fewer than 40% of items were missing for that scale. Missing items were left as missing if they were not part of a scale or if a questionnaire was not returned.

Participation and response rates are presented by arm and the characteristics of participating and non-participating schools are described. School and girl demographic characteristics are presented by arm.

The proportion of missing datum for consent form return, vaccination status, IMD and ethnicity are described. Multivariable-logistic regression was used to explore predictors of girls and parents missing at least one questionnaire item, among those who returned a questionnaire.

Consent form return rates and vaccination uptake are presented by arm, with 95% CIs for the proportions where the standard errors were adjusted for clustering using the ‘vce’ command in STATA (StataCorp, College Station, TX, USA). Possible unintended consequences of the incentive are presented by arm, along with possible mechanisms of action, with 95% CIs for the proportions where the standard errors were adjusted for clustering using the ‘vce’ command in STATA as before.

A trial management group was consulted at the beginning and end of the study. It was determined that a data monitoring committee was not required or interim analyses or stopping guidelines. Ethical approval was received from the UCL Research Ethics Committee (6615/002) and the trial protocol was registered (ISRCTN72136061).

## Results

### Feasibility outcomes

**Figure 1:**
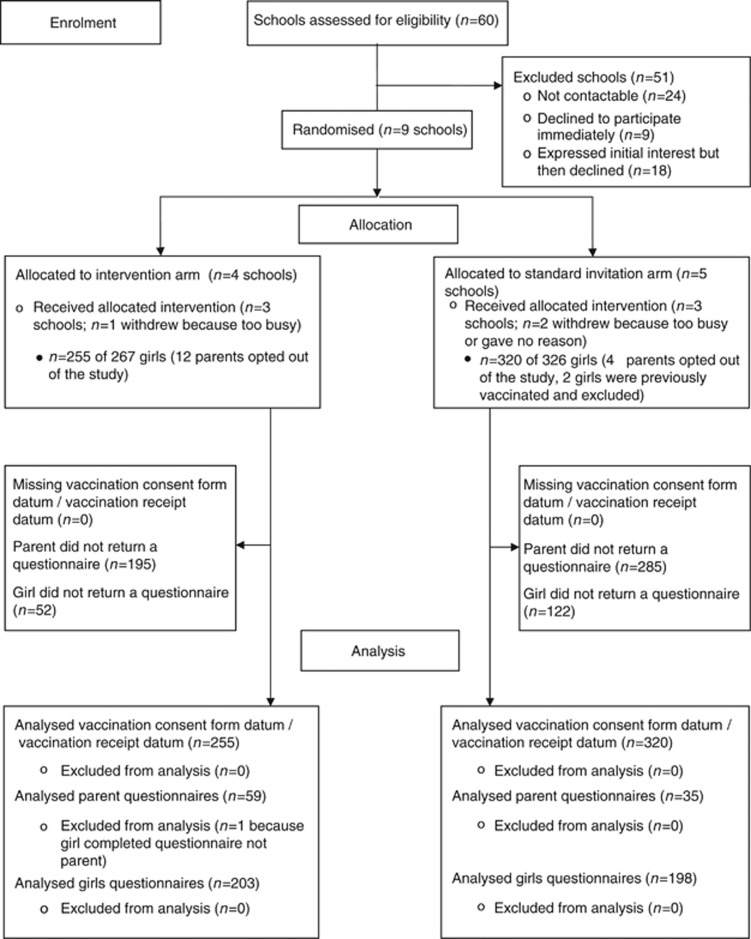
**Trial flow diagram.**

There were 60 eligible schools in the participating boroughs, all of whom we approached; 24 of these schools were not contactable on the phone and/or did not respond to an email, nine declined to participate immediately and 18 expressed an interest in the study, but then declined. Most of these schools did not provide a reason for their non-participation, but for those that did, reasons related to lack of capacity rather than objections to the trial itself. We randomised nine schools (four intervention arm, five standard invitation arm). Two schools withdrew because they were too busy, one in each arm and one withdrew without providing a reason (standard invitation arm). Six schools completed the trial (10% three Enfield, three Southwark). In participating schools, 16 out of 593 parents opted out of the study (3%) and 2 girls were excluded from analyses because they had received the vaccine previously. Among parents of participating girls, 95 out of 575 returned a questionnaire (17%) and 401 out of 575 girls completed a questionnaire (70%). Response rates by trial arm are reported in Supplementary Material (Supplementary Table S1). See Figure 1 for trial flow diagram.

Around 83% (*n*=5) of participating schools were co-educational and 82% (*n*=44) of non-participating schools. Of participating schools, around 67% (*n*=4) had no religious affiliation, as did 76% (*n*=41) of non-participating schools. The median school size for participating schools was 1013 and 817 for non-participating schools.

Three schools were randomised into each arm (intervention arm: *n*=255 girls; standard invitation arm: *n*=320 girls; Table 1). All schools in in the intervention arm were co-educational as were two schools in the standard invitation arm. Two schools in each arm had no religious affiliation. School size in the intervention arm ranged from 828–1432 and from 263–1071 in the standard invitation arm. Girls’ ethnicity data were largely missing because this item was ascertained from parents’ questionnaires (available for 15% of girls). Of responders, the most represented ethnic group in the intervention arm was White British and in the standard invitation arm was African or ‘Other’ ethnic groups. In both arms the most commonly reported religion was Christian. Girls in the intervention arm reported a mean religiosity score of 3.8 (range 1–7) and this was 4.9 in the standard invitation arm. Most girls in both trial arms lived in areas in the most or second most deprived quintile for England. A large proportion of girls in both arms were born in the United Kingdom, but their parents were not.

There was no missing datum for whether a consent form was returned by participating girls, whether consent for vaccination was received or IMD quintile. Multivariable-logistic regression showed that having missing data in a returned questionnaire was not associated with trial arm and IMD for both parents and girls and also with school for girls (numbers were too small to assess relationships between missing data and school for parents; see Supplementary Material Supplementary Table S2).

### Proof-of-concept outcomes

The proportion of consent forms returned was 87% in the intervention arm (*n*=222) and 67% (*n*=215) in the standard invitation arm. The proportion of girls who gained consent to receive the vaccine was 76% (195) in the intervention arm and 61% (196) in the standard invitation arm (Table 2). There was wide variation between schools for these outcomes, with particularly low consent form return rates and vaccination receipt among girls in School Four (standard invitation arm).

Possible unintended consequences of the intervention are reported in Table 3. Around 52% of parents in the intervention arm made an informed decision about HPV vaccination for their daughter (i.e., they had good knowledge and their attitudes were consistent with whether their daughter received the vaccine or not), and this was 27% in the standard invitation arm. Around 71% of parents in both arms agreed or strongly agreed that incentives to maximise consent form return are a good idea. Only 8% of girls in the intervention arm said they would not return a future consent form without incentive (16 of 198 girls; 15 of these had returned a consent form in the trial).

Possible mechanisms of action of the intervention are reported in Table 3. Around 77% of girls in the intervention arm found it easy to remember to return their consent form compared with 69% of girls in the standard invitation arm. When asked whether returning the consent form was important, 62% of girls in the intervention arm and 68% of girls in the standard invitation arm thought it was. In both arms, around 53% of girls were motivated to return their consent form. Around 70% of girls in the intervention arm gave their parents the consent form the same day they received it from school compared with 58% of girls in the standard invitation arm. Among girls in the intervention arm, 89% of those with a low FOMO score (indicating less FOMO) returned their consent form, compared with 93% of girls with a high FOMO score.

## Discussion

We have demonstrated the feasibility of conducting a cluster RCT of an incentive intervention to improve HPV vaccination uptake among girls. Ten percent of schools participated and very few parents of girls within these schools opted out of the trial. Complete data for the main future trial outcomes were collected and we obtained high response rates to a questionnaire completed by girls. It was not feasible to collect data from parents via a questionnaire as response rates were low. The preliminary results showed that vaccination consent form return rates were 20 percentage points higher in the intervention arm compared to the standard invitation arm and HPV vaccination rates were 15 percentage points higher in the intervention arm compared to the standard invitation arm.

The between-arm differences for the proof-of-concept outcomes were large; 20 percentage points for consent form return rates and 15 percentage points for vaccination consent rates. However, lower consent form return rates and vaccination consent rates in the standard invitation arm may have been due to particularly low rates in School Four. Differences between groups may have been due to the anomaly of this school rather than any intervention effect. However, consent form return rates and vaccination rates were still lower in the standard invitation arm if School Four was removed from this analysis. A different interpretation of these data is that there was a greater proportion of girls from ethnic minority groups in the standard invitation arm, and it is known that HPV vaccine uptake is lower among girls from these backgrounds (Fisher *et al*, 2013a,b; Bowyer *et al*, 2014). However, findings related to ethnicity must be interpreted with caution because of the high proportion of missing data. The result highlights the importance of any future trial having a large number of clusters to balance such discrepancies across arms. Vaccination rates in this trial were lower than the borough averages for the preceding academic year; dose one in Enfield in 2015/16 was 82% and 90% in Southwark (Public Health England, 2016). It is likely to be that efforts by immunisation teams throughout the academic year to ‘mop-up’ those who were unvaccinated at the first immunisation session have increased vaccination rates. Our preliminary data suggests that an incentive intervention might be able to improve vaccination uptake, and such an intervention might help reduce the workload of immunisation nurses. This is likely to be the case without negative consequences, as a higher proportion of parents of daughters in the intervention arm made an informed decision about HPV vaccination, compared with the standard invitation arm, and parents in both arms thought that incentives were a good idea. Only a small minority of girls thought they would not return future consent forms without incentive.

Preliminary data suggest that the incentive may work by helping girls to remember to return their consent form and girls in the intervention arm were more likely to give their parents the consent form to sign on the day that they received it from school. This is in line with evidence from neuroscience, which suggests that reward-related brain activation is linked to improved memory (Kang *et al*, 2009), whereas others have suggested that incentives result in individuals allocating cognitive capacity towards obtaining the reward (Marteau, 2010). Among girls in the incentive arm, there was also an indication that those who were more likely to fear missing out on something their peers were experiencing were more likely to return the consent form. Little is known about this phenomenon (Przybylski *et al*, 2013), particularly among young people, and deserves further attention.

The combined feasibility outcomes indicate that an RCT of the incentive intervention is feasible. Ten percent of schools agreed to participate, which is lower than other comparable school-based cluster RCTs (e.g., Henderson *et al*, 2007; Starkey *et al*, 2005). However, the first of these trials provided incentives for school participation and the latter conducted an anti-smoking intervention, which may have been perceived to be of greater relevance to their pupils. Participating schools in this trial may differ from those that did not participate, limiting the generalisability of the trial findings. This participation rate estimate will help researchers to plan recruitment for a larger RCT. It will be important to ensure that participating schools in a future trial are representative of schools in England, potentially by stratifying randomisation by school characteristics and vaccination uptake from the previous year. Obtaining consent for participation from local immunisation teams and local authorities that oversee schools, rather than approaching individual schools directly, may result in a more representative sample of schools participating. Few parents opted out of the study and the majority of girls completed questionnaires, with a high proportion of complete data among those who did so. Response rates to the parent questionnaire were not optimal and suggest that collecting data from parents in this way is not feasible. When designing a future RCT, other approaches to obtain these data will need to be explored, such as the use of online questionnaires, emailed to parents by participating schools. Further efforts could be made to increase response rates, for example it is known that financial incentives up to the value of $5 improve questionnaire response rates (Edwards *et al*, 2005; Edwards *et al*, 2009), as does providing non-responders with a second copy of the questionnaire (Edwards *et al*, 2009); however, these methods are still unlikely to bring response rates up to an optimal level. The low parental response rate resulted in incomplete information on girls’ ethnicity. Such information will be essential for a future RCT to monitor effects on ethnicity. In a future RCT information on girls’ ethnicity may be better collected from girls themselves, rather than by proxy. It is difficult to comment on the reason behind low response rates among parents; however parental response rates were low among schools that also had low response rates for the girls’ questionnaire.

In line with guidance on conducting randomised feasibility trials (Eldridge *et al*, 2016), the study was not powered to detect differences between groups. Evidence of efficacy will only be provided by a future RCT. It was not possible to obtain datum on HPV vaccination uptake by school for the year prior to this study, so we do not know if participating schools were those that previously had low or high vaccination uptake. The low parental response rate reduces any certainty we might have about findings relating to this questionnaire. The impact of the intervention on informed decision-making must be monitored in a future RCT.

The findings of this cluster randomised feasibility trial demonstrate that a future RCT of an adolescent incentive intervention to improve HPV vaccination uptake is feasible, and preliminary data suggest that the intervention may improve consent form return rates and vaccination uptake. Testing of efficacy on a larger scale is required, further exploring potential mechanisms of action of the incentive, including whether the incentive makes girls more likely to remember to return their consent form. It will be important also to observe whether the incentive intervention continues to result in no unintended negative consequences. This incentive intervention has the potential to substantially improve HPV vaccination uptake, which should reduce HPV-related cancer incidence, with minimal work from immunisation providers.

## Figures and Tables

**Table 1:** School characteristics and girls’ demographic characteristics

	**Intervention arm**		**Standard invitation arm**	
**School characteristics (*n*=6)**				
Single sex *n (%)*	0	(0.0)	1	(33.3)
School religion *n (%)*				
Christian	1	(33.3)	1	(33.3)
None	2	(66.7)	2	(66.7)
**Girls’ demographics (*n*=575)**				
Ethnicity^a^ *n (%)*				
White British	29	(49.2)	10	(28.6)
African	9	(15.3)	12	(34.3)
White Other	5	(8.5)	1	(2.9)
Other	16	(27.1)	12	(34.3)
Missing *n*	197	—	285	—
Religion^b^ *n (%)*				
None	65	(33.0)	5	(2.6)
Christian	101	(51.3)	158	(83.2)
Muslim	22	(11.2)	17	(9.0)
Other	9	(4.6)	10	(5.3)
Missing *n*	58	—	130	—
Religiosity^b^ Mean (s.d.) range 1–7	3.8	(2.2)	4.9	(1.5)
IMD quintile^c^^,^^d^ *n (%)*				
Most deprived: 1	150	(58.8)	230	(71.9)
2	43	(16.9)	64	(20.0)
3	28	(11.0)	18	(5.6)
4	14	(5.5)	7	(2.2)
Least deprived: 5	20	(7.8)	1	(0.3)
Missing *n*	0	—	0	—
Migration status^b^ *n (%)*				
Girl born UK, parents born UK	52	(29.1)	31	(17.4)
Girl born UK, 1 parent born UK	23	(12.9)	17	(9.6)
Girl born UK, neither parent born UK	86	(48.0)	102	(57.3)
Girl not born UK, neither parent born UK	16	(8.9)	28	(15.7)
Girl not born UK, 1 parent born UK	1	(0.6)	0	(0.0)
Girl not born UK, parents born UK	1	(0.6)	0	(0.0)
Missing *n*	76	—	142	—

a Data source: Parents’ questionnaire.

b Data source: Girls’ questionnaire.

c Data source: Collected from schools.

d Quintile for the whole of the United Kingdom.

**Table 2:** Consent form return rates and whether consent was given for vaccination by school and trial arm (*n*=575)

	**School number/arm**							
	**Intervention arm**			**Standard invitation arm**				
	**1**	**2**	**3**	**4**	**5**	**6**	**Intervention arm** ***n/N*** **(%, 95% CI)**	**Standard invitation arm** ***n/N*** **(%, 95% CI)**
Consent form returned	66 (97.1)	76 (100.0)	80 (72.1)	38 (39.6)	139 (78.5)	38 (80.8)	222/255 (87.1, 47.5–98.0)	215/320 (67.2, 34.6–88.8)
Consent given for vaccination	62 (91.2)	67 (88.2)	66 (59.5)	35 (36.5)	130 (73.5)	31 (65.0)	195/255 (76.5, 44.0–93.1)	196/320 (61.3, 32.4–83.9)

**Table 3:** Possible unintended consequences, mechanisms of action and acceptability of the incentive

	**Intervention arm** ***n/N*** **(%, 95% CI)**	**Standard invitation arm** ***n/N*** **(%, 95% CI)**
**Possible unintended consequences**		
Parents made an informed decision	28/54 (51.9, 26.7–76.1)	9/33 (27.3, 7.6–63.2)
**Acceptability**		
Incentives are a good idea	42/59 (71.2, 47.7–87.0)	25/35 (71.4, 45.0–88.4)
**Possible mechanisms of action**		
Very/quite motivated to return the form	105/200 (52.5, 35.0–69.4)	101/191 (52.9, 45.7–59.9)
Very easy/easy to remember to return the form	154/201 (76.6, 43.5–93.3)	133/193 (68.9, 63.6–73.8)
Returning the form is very/quite important	125/203 (61.6, 39.7–79.6)	131/194 (67.5, 59.1–74.9)
Gave consent form to parents the same day they received it from school	141/202 (69.8, 47.6–85.5)	111/193 (57.5, 52.3–62.5)
