# Supplementary material for: A cluster randomised feasibility study of an adolescent incentive intervention to increase uptake of HPV vaccination
Source: Br J Cancer. 2017 Aug 22;117(8):1121–7. doi: 10.1038/bjc.2017.284 (PMC5674104; doi:10.1038/bjc.2017.284)
Supplement: Supplementary Material [file bjc2017284x1.docx]

***Supplementary material***

**Measures used in the study**

*Mechanisms of action of the intervention*

The incentive increasing motivation to return the consent form - ‘When you were given the HPV vaccine consent form, how motivated were you to get it signed and return it?’, very motivated-very unmotivated, five-point scale, dichotomised to ‘very or quite motivated’ versus other responses in analysis)

The incentive improving memory to return the consent form (‘How easy was it for you to remember that you needed to return the consent form?’, very easy-very hard, five-point scale, dichotomised to ‘very or quite easy’ versus other responses in analysis)

The incentive increasing the salience for returning the consent form (‘When did you give the consent form to your parent / guardian?’, I gave it to them as soon as I got home-I never gave it to them, seven response options, dichotomised to ‘consent form given to parents the day the girl received it’ versus other responses in analysis)

The incentive increases the perceived value of returning the consent form (‘How important was it to you to return the consent form?’, very important-very unimportant, five-point scale, dichotomised to ‘very or quite important’ versus other responses in analysis)

Fear of missing out ([FOMO, using Przybylski *et al*, 2013](#_ENREF_30)), categorised as high or low FOMO, split at the median=3.2 in analysis).

*Unintended consequences*

Girls (incentive arm only)

‘In the future, would you return a vaccination consent form if you were not entered into a prize draw to win a £50 voucher?’, yes, definitely-no, definitely not, five-point scale).

Parents

‘I think it is a good idea for girls to be entered into a prize draw to win a £50 voucher if they return their HPV vaccine consent form’, strongly agree-disagree, five-point scale)

Informed decision-making as described by Mantzari et al. ([Mantzari *et al*, 2015](#_ENREF_22)).

*Demographics*

Girls

Religion ([based on Office for National Statistics, 2011](#_ENREF_28))

Strength of any religious faith ([European Social Survey](#_ENREF_11))

Migration status (whether they and their parents were born in the UK; adapted from ([Marlow *et al*, 2015](#_ENREF_23))).

Parents

Daughter’s ethnicity ([using Office for National Statistics, 2011](#_ENREF_28)).

*Table S1 - Participation / response rates by and arm*

|  | **Intervention arm**  n/N  (%, 95% CI) | **Standard invitation arm**  n/N  (%, 95% CI) |
| --- | --- | --- |
| Parents opting-out | 12/267  (4.5, 3.2-6.3) | 4/326  (1.2, 0.6-2.5) |
| Parents’ questionnaire response rate | 60/255  (23.5, 9.0-49.0) | 35/320  (10.9, 6.2-18.7) |
| Girls’ questionnaire response rate | 203/255  (79.6, 41.6-95.5) | 198/320  (61.9, 12.9-94.7) |

*Table S2 - Demographic predictors of having at least 1 missing item on questionnaire among responders*

| **Parent questionnaire** | **No missing data**  *n (%) ^a^*  47 (49.5) | **Missing data**  *n (%) ^a^*  48 (50.5) | **OR**  (95% CI) | **Adjusted OR**  (95% CI) |
| --- | --- | --- | --- | --- |
|  |  |  |  |  |
| IMD quintile  mean (SD)^b^ | 2.5 (1.4) | 1.5 (1.0) | 0.8 (0.6-1.0) | 0.9 (0.5-1.4) |
| Arm |  |  |  |  |
| Intervention | 32 (68.1) | 28 (58.3) | 0.7 (0.3-1.5) | 1.1 (0.2-5.1) |
| Standard invitation | 15 (31.9) | 20 (41.7) | Ref. | Ref. |
|  |  |  |  |  |
| **Girl**  **questionnaire** | **No missing data**  *n (%) ^a^*  287 (71.6) | **Missing data**  *n (%) ^a^*  114 (28.4) | **OR**  (95% CI) | **Adjusted OR**  (95% CI) |
|  |  |  |  |  |
| IMD quintile  mean (SD) ^b^ | 1.7 (1.1) | 1.5 (0.9) | 1.0 (0.8-1.2) | 1.2 (0.9-1.6) |
| Arm |  |  |  |  |
| Intervention | 147 (51.2) | 56 (49.1) | 0.9 (0.6-1.4) | 1.5 (0.4-5.9) |
| Standard invitation | 140 (48.8) | 58 (50.9) | Ref. | Ref. |
| School |  |  |  |  |
| 1 | 47 (16.4) | 16 (14.0) | Ref. | Ref. |
| 2 | 55 (19.2) | 17 (14.9) | 0.9 (0.4-2.0) | 0.6 (0.2-1.7) |
| 3 | 45 (15.7) | 23 (20.1) | 1.5 (0.7-3.2) | 1.5 (0.7-3.1) |
| 4 | 14 (4.9) | 8 (7.0) | 1.7 (0.6-4.7) | 2.5 (0.5-11.3) |
| 5 | 113 (39.4) | 47 (41.2) | 1.2 (0.6-2.4) | 1.7 (0.5-6.3) |
| 6 | 13 (4.5) | 3 (2.6) | 0.7 (1.7-2.7) | 1.0 |
|  |  |  |  |  |

^a^ Unless specified

^b^ Possible range: 1-5
